# Subjective health awareness and sensory ability of taste and olfaction: A case study of a health promotion class for older people

**DOI:** 10.1371/journal.pone.0275093

**Published:** 2022-10-10

**Authors:** Sana Inoue, Junji Watanabe, Yuji Wada

**Affiliations:** 1 BKC Research Organization of Social Sciences, Ritsumeikan University, Kusatsu, Shiga, Japan; 2 NTT Communication Science Laboratories, Nippon Telegraph, and Telephone Corporation, Atsugi, Kanagawa, Japan; 3 College of Gastronomy Management, Ritsumeikan University, Kusatsu, Shiga, Japan; Université de Bourgogne: Universite de Bourgogne, FRANCE

## Abstract

The quality of the dietary habits of older adults is important for increasing healthy life expectancy. As with other physical senses, the senses of taste and olfaction change with age. In contrast to physical sensations that can be visibly compared with those of other people, taste and olfaction are personal sensations, making it challenging to infer associated changes. This study investigated the characteristics of taste and olfaction in healthy older adults and compared them with those of young adults. In the taste assessment, threshold values were measured using the whole-mouth method with a diagnostic assay kit (Tastedisc). The olfactory assessment measured the overall identification ability using a card-type olfactory identification test kit (Open Essence). Additionally, participants’ subjective health awareness was measured using a visual analog scale. The taste and olfactory assessments results showed that the older group had lower overall sensory sensitivity than the young group, and that there was no correlation between taste and olfactory sensitivity in the older group, while a trend was observed in the young group. Moreover, there was no significant difference between the two groups regarding subjective health awareness, indicating that participants in our research considered themselves healthy regardless of age. This suggests that the subjective health awareness of older people in the health promotion class is somehow independent from their sensory ability.

## Introduction

Dietary habits are important in the maintenance and improvement of an individual’s health. Taste and olfaction are essential for food consumption. Constant difficulties associated with these sense perceptions may hamper the ability to derive satisfaction and well-being from eating behavior, reducing an individual’s intake of necessary nutrients due to decreased appetite [1–3]. Multiple factors have been identified for these taste and olfactory disorders, including the effects of a disease, side effects of medications [3,4], and aging. In the case of taste, there are basic taste qualities, such as sweet, salty, sour, and bitter, and each of these exhibits different sensitivity changes due to aging (e.g., [5]). Additionally, a decline in subjective taste often reflects an impairment in the actual olfactory function [6], and taste and olfaction are closely related. Decreased olfaction has been reported to be associated with Alzheimer’s disease, a common disease among older adults [7,8]. As olfactory function acts as one of the strongest predictors of five-year mortality, it also serves as a predictive indicator of life expectancy [9]. These findings indicate that the sense of taste and olfaction are important aspects that can act as a health barometer for older adults. While the objective health assessment indicates qualitative health, subjective health awareness is a self-assessment of sensory perception. Mossey and Shapiro (1982) reported that older adults with lower subjective health awareness had a higher mortality rate within six years than those with higher subjective health awareness as a predictor independent of objective health [10]. Subsequent studies have also shown that subjective health awareness was associated with the risk for mortality. Idler and Benyamini (1997) indicated that subjective health awareness was a vital health index [11]. Therefore, it is essential to understand the actual relationship between subjective health awareness, subjective/actual sense of taste, and olfaction in healthy older adults, which may help maintain their quality of life and health.

Although there have been several medical studies on taste or olfactory impairment in older adults in Japan (e.g., [12]), fewer comprehensive studies on these sensations have been conducted among healthy older adults (e.g., [13]). The present study focused on the relationship between subjective health awareness and qualitative taste and olfaction in older adults compared to young adults from the perspective of health maintenance.

## Materials and methods

### Research policy and ethics

Research on older adults needs to be conducted more carefully compared to general adult subjects. Particular attention must be paid to safety management, and older adults, even if they are healthy, are at greater risk for accidents due to their advanced age. This study considered a health promotion class for older people at a city-run study facility for healthy older adults aged 60 years and above. Participants could join the experiments in the facility between the lectures they were taking. Minimal distance and measurement in a familiar environment minimized the burden on the participants. Additionally, because the older adults were expected to vary from person to person during the experiment, each participant was assigned a research assistant in case of an emergency. A briefing session was held for the assistants before the survey to explain the study, its flow, and the methods for emergency response. Upon receiving permission to use the facilities, older students who had enrolled in two classes offering lectures on health were given presentations that explained the purpose of the study. Participants were recruited after their informed consent was obtained. For the group of young adults, which acted as a comparison for the group of older adults, students between 18 and 25 years of age who did not require parental consent were recruited. They participated in the experiment at university campuses, where they usually attended lectures. All participants were required to read an explanation of the purpose of the study. Further, they were required to sign a paper consent form before the experiment. The experiment was conducted with those who agreed to participate and signed a consent form. Before the recruitment of the research participants, an ethics review was performed at the Ritsumeikan University (Kinugasa-Human-2017-15). The study was conducted per the code of ethics set by the Declaration of Helsinki and all of its amendments.

### Participants

There were 56 healthy participants (16 men and 40 women, median age: 73 years [interquartile range, 71–75 years]) in the older adult group and 55 healthy participants (18 men and 37 women, median age: 19 years [interquartile range, 19–21]) in the young adult group.

### Procedure

The experiment consisted of three tasks. The performance of each task was carried out at the participants’ own pace, and after the task was completed, the participant proceeded to the next task. For the older adult group, one research assistant per participant was in attendance during each task.

#### Subjective health awareness

A questionnaire was administered to determine the participants’ subjective awareness of their health. A visual analog scale (VAS), a scale that measures aspects that cannot be directly measured, such as psychological state, was used. Participants were asked to respond to the following four questions ranging from “very bad” at the left end of a 10 cm straight line to “very good” at the right end.

- How is your current general health status?
- How is your general health at present compared to a year ago?
- Are you generally sensitive to differences in taste in your current state?
- Are you generally sensitive to differences in smell in your current state?

#### Taste assessment

A whole-mouth gustatory test was applied, which can diagnose taste quality, along with Tastedisc®, a diagnostic assay kit for taste assessment using filter-paper disks (Sanwa Kagaku Kenkyusho Co., Ltd., Nagoya, Aichi, Japan) [14]. Previous studies have compared the original use of this assay with the filter-paper disk method and the whole-mouth gustatory test, and positive better-than-moderate correlations were reported between the two methods [15,16]. The whole-mouth gustatory test was chosen for the present study because of its low stress load on the participants. The Tastedisc consisted of five reagent concentrations for each of the four taste qualities: sweet, salty, sour, and bitter. The sweet liquid was composed of refined white sugar (0.3%, 2.5%, 10%, 20%, and 80%), the salty liquid was sodium chloride (0.3%, 1.25%, 5%, 10%, and 20%), the sour liquid was tartaric acid (0.02%, 0.2%, 2%, 4%, and 8%), and the bitter liquid was quinine hydrochloride (0.001%, 0.02%, 0.1%, 0.5%, and 4%) [14].

The sweet, salty, and sour taste liquids were presented in a pseudo-random order following the standard method. The bitter taste liquid was always last, and each individual taste was presented in ascending order from lower to higher concentrations. However, to prevent aspiration, participants were required to use a dropper to drip each sample in their mouths by themselves while checking a mirror. Participants were asked to first rinse their mouth with water, then drop a spot of the liquid in the center of the tip of their tongue, and finally taste it with their entire tongue. In response, the participants pointed to one of the options given on the taste quality index (sweet, salty, sour, bitter, unknown but some taste, or tasteless). The concentration was increased step-by-step until the participants answered correctly.

#### Olfactory assessment

In the olfactory test, Open Essence®, a card-type olfactory identification test kit designed for the Japanese (FUJIFILM Wako Pure Chemical Corp., Osaka, Japan) [17], was used. The kit consisted of 12 typical household aromas that were familiar to the Japanese: perfume, rose, condensed milk, mandarin orange, curry, roasted garlic, musty socks, household gas, menthol, sumi ink, lumber, and Japanese cypress [18,19]. The aroma was applied to the inside of each card’s facing page. The inner capsule popped open when the card was opened, and the aroma could be perceived, although the odor did not diffuse into the room and dissipated quickly [17]. This diagnostic kit for clinical research can measure overall olfactory identification ability based on the total number of correct answers. Further, it is suitable for studies with older adults, as it has been reported to be valuable for screening for age-related changes in olfactory function in previous studies [18,20].

Participants opened the cards individually in a specific order from card A to L, smelled them, and then selected one of the six alternatives that included the correct answer, three other aromas, “I do not know,” and “Odorless.”

## Results

Data analyses were conducted using the statistical software HAD ver. 16_057 [21].

### Subjective health awareness

**Fig 1:**
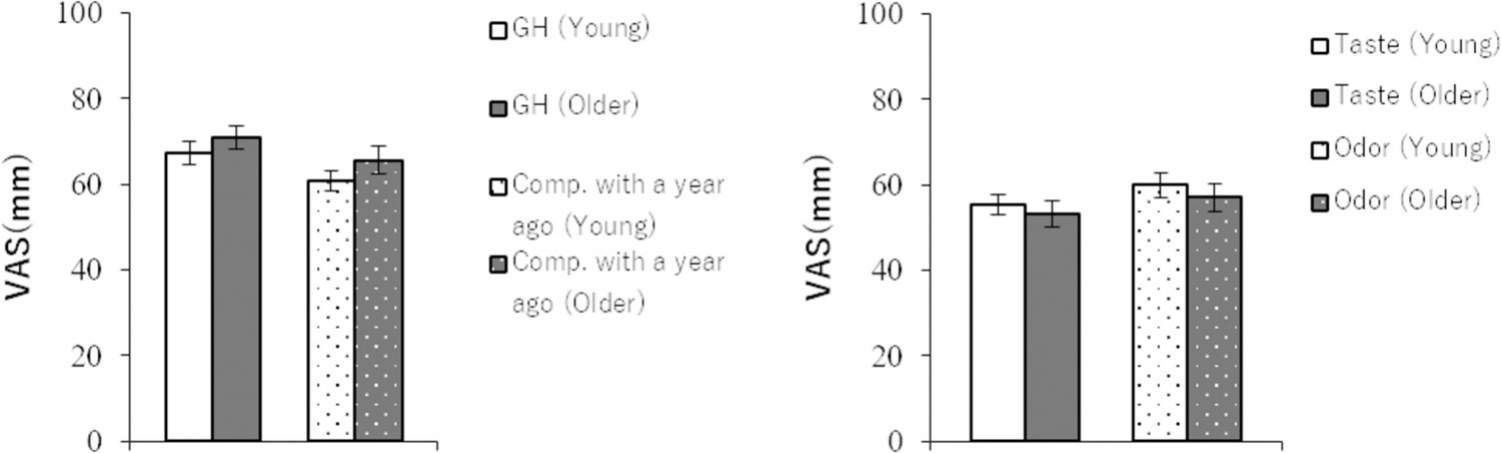
**a. Subjective current status of general health and comparison with the previous year.** Error bars are 95% confidence intervals. **b. Subjective current sensitivity of taste and olfaction.** Error bars are 95% confidence intervals.

T-tests were conducted, and there were no significant differences between the two groups in any of the four questionnaires: a) current general health status, older adults 70.89±2.65 mm, young adults 67.29±2.59 mm (t(109) = 0.97, p = .33); b) general health at present compared to a year ago, older adults 65.63±3.12 mm, young adults 60.77±2.51 mm (t(109) = 1.21, p = .23); c) general sensitivity of taste, older adults 53.32±3.07 mm, young adults 55.46±2.37 mm (t(109) = 0.55, p = .58); and d) general sensitivity of olfaction, older adults 57.12±3.25 mm, young adults 59.94±2.88 mm (t(109) = 0.65, p = .52) (Fig 1).

### Taste assessment

**Fig 2:**
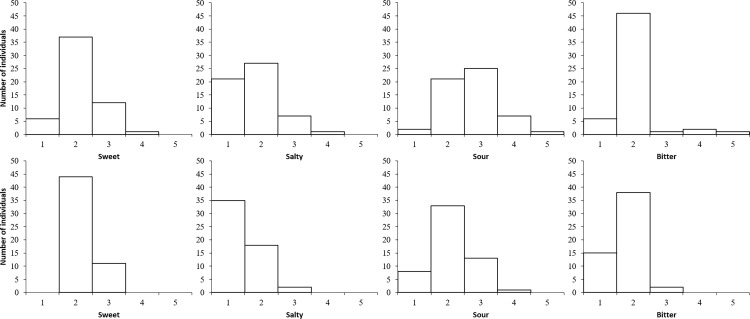
Distribution of taste threshold (upper column: Older adults; lower column: Young adults). The horizontal axis indicates the density of the concentration (1: Lowest, 5: Highest). Thus, a lower concentration level indicates higher taste sensitivity and vice versa.

The taste thresholds of both groups were determined from the responses to the concentrations of each taste quality (sweet, salty, sour, and bitter) (Fig 2). For each taste quality, the concentration at which the responses were correct was defined as the cognitive threshold. Taste sensitivity level 1 (the lowest level) to level 5 (the highest level) was indicated by the respondent. The sensitivity decreased as the number increased from concentration level 1, which represented the highest taste sensitivity, to level 5, which represented the lowest sensitivity, for sour and bitter tastes for some older adults.

**Fig 3:**
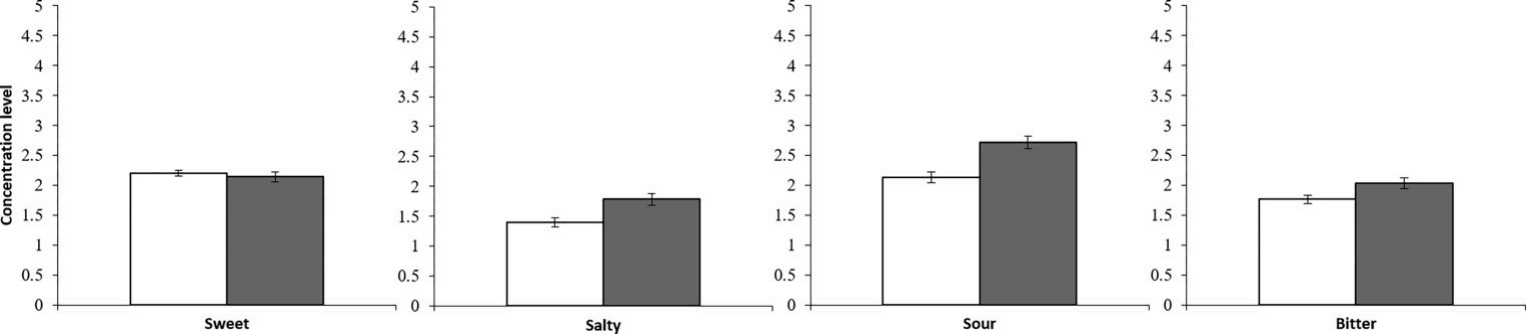
Concentration levels of each taste in the young group (white) and older group (gray). Error bars are 95% confidence intervals.

The overall taste function between the older and young groups was compared using the total score of the concentration levels of the four taste qualities. A t-test revealed that the older group needed a higher concentration level to produce a correct answer than the young group (older adults 8.5±0.3, young adults 7.5±0.2, t(109) = 3.19, p < .01). In the analysis of each taste, the concentration levels were counted as scores, and t-tests showed no significant differences in the sweet taste category. However, significant differences were revealed for the other taste qualities. The older group required a higher concentration level for correct answers compared to the young group (sweet: older adults 2.1±0.1, young adults 2.2±0.1, t(109) = 0.58, p = .57; salty: older adults 1.8±0.1, young adults 1.4±0.1, t(109) = 3.11, p < .001; sour: older adults 2.7±0.1, young adults 2.1±0.1, t(109) = 4.18, p < .001; and bitter: older adults 2.0±0.1, young adults 1.8±0.1, t(109) = 2.43, p < .05; Fig 3).

### Olfactory assessment

**Fig 4:**
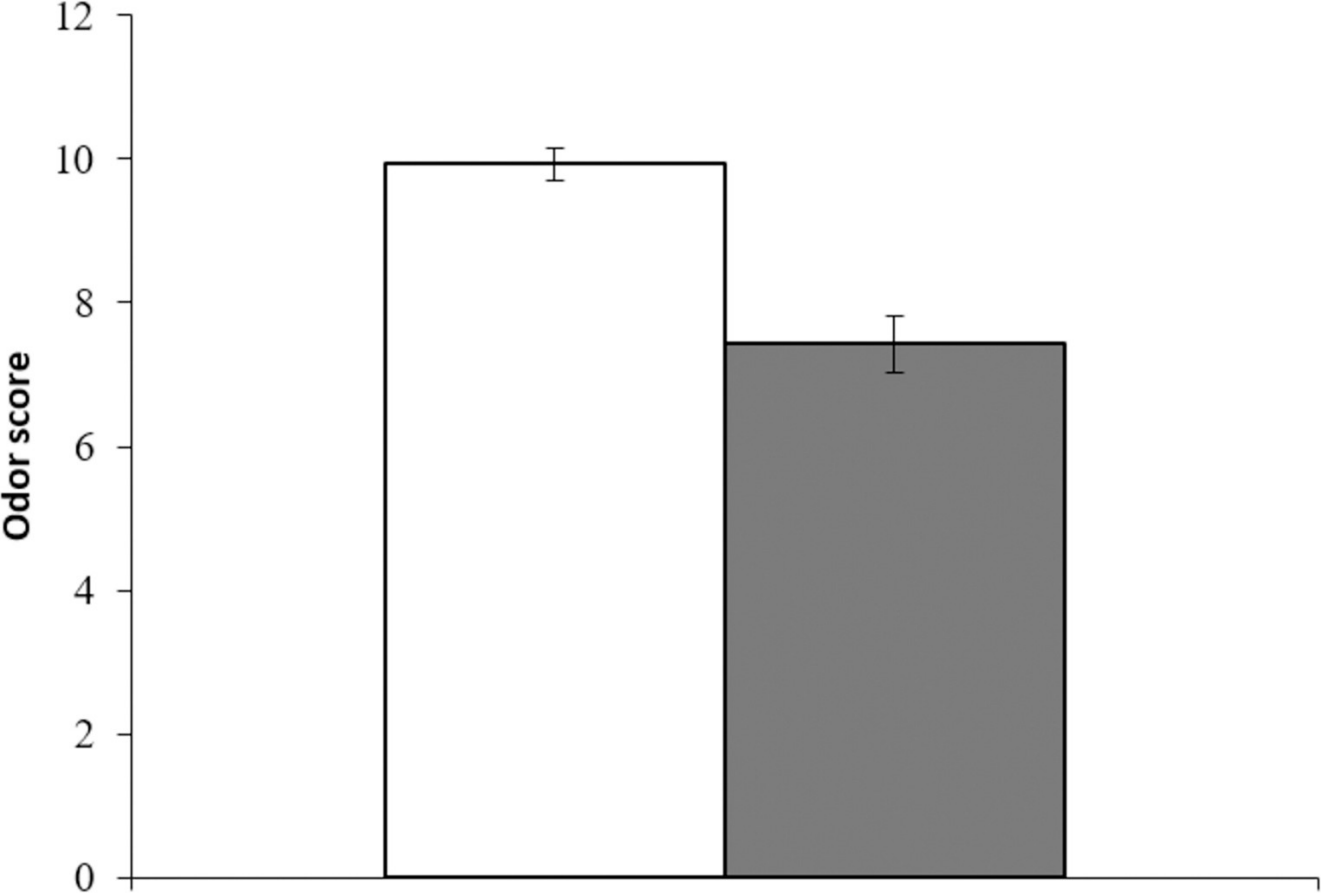
Odor scores for the young group (white) and older group (gray). Error bars are 95% confidence intervals.

The older group showed significantly lower odor scores (maximum 12 points) than the young group (older adults 7.4±0.4, young adults 9.9±0.2, t(109) = 5.58, p < .001; Fig 4). Based on the cutoff score of eight or less as a reference point for olfactory deficiency used in previous studies [18,22], the mean score of the older group was classified as deficient, which was sufficient to indicate a “poor” condition. From the comparison of the rank scores for each odor, there was a significant difference between the two groups for the nine aromas (lumber: older adults 51.2, young adults 60.9, z(1) = 2.07, p < .05; perfume: older adults 49.7, young adults 62.4, z(1) = 2.74, p < .01; menthol: older adults 45.2, young adults 67.0, z(1) = 4.62, p < .001; mandarin orange: older adults 44.8, young adults 67.4, z(1) = 4.38, p < .001; curry: older adults 52.6, young adults 59.5, z(1) = 2.39, p < .01; rose: older adults 47.3, young adults 64.9, z(1) = 3.51, p < .001; musty socks: older adults 47.7, young adults 64.5, z(1) = 3.79, p < .001; condensed milk: older adults 50.7, young adults 61.4, z(1) = 2.31, p < .05; and roasted garlic: older adults 50.2, young adults 61.9, z(1) = 2. 55, p < .05). For all of these aromas, including Japanese cypress (older adults 52.1, young adults 59.9, z(1) = 1.78, p = .07), which showed a marginal difference, the older group had lower scores. However, no significant differences were found for the remaining two aromas (sumi ink: older adults 55.7, young adults 56.3, z(1) = 0.11, p = .91) and household gas (older adults 56.2, young adults 55.8, z(1) = 0.06, p = .95).

### Correlation between taste and olfaction

**Fig 5:**
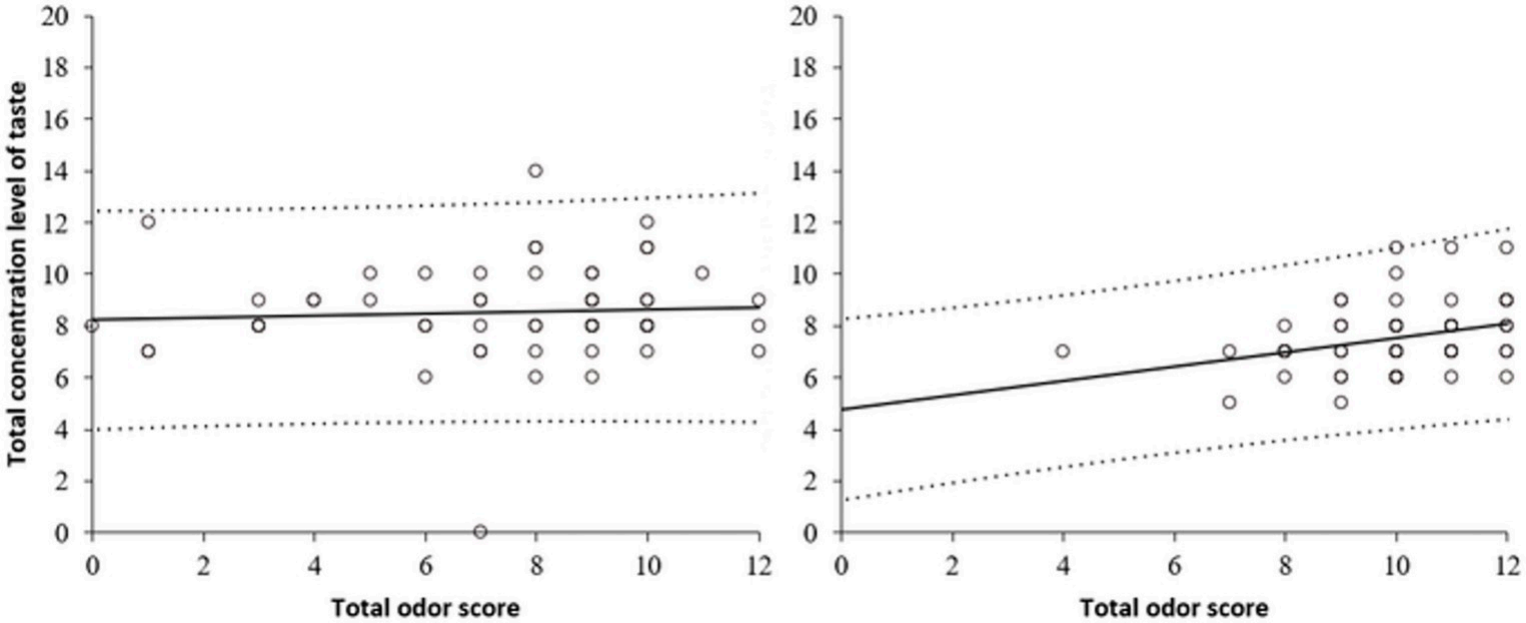
Linear fit between taste and olfaction in older adults (left) and young adults (right). The dots indicate data points, and dashed lines indicate the 95% prediction intervals.

Based on the results of taste and olfactory assessments, the relationship between taste and olfaction was analyzed. A regression analysis was conducted to determine the correlation between the total number of taste concentrations and odor scores in each group (Fig 5). No significant differences were found in the older group (β = 0.60, p = .66, R2 = 0.004), whereas in the young group, higher concentration levels were associated with higher odor scores (β = 0.32, p < .05, R2 = 0.10).

## Discussion

This study focused on a comprehensive investigation of taste and olfaction. It involved experiments using the four basic taste qualities and 12 household odors, such that the sensations were close to those experienced in everyday situations. The taste and olfactory assessment results showed that the older group had lower overall sensory sensitivity than the young group. As with previous studies (e.g., [23]), variations in sensitivity were observed for each of the four taste qualities examined, suggesting an effect of aging rather than a uniform decline in taste in older adults. In olfaction, the older group not only scored lower than the younger adults but also showed a lower mean value, which is classified as olfactory impairment. This was despite the fact that the odors used for assessment were ones that older adults were likely to be exposed to in their daily life. Taking into account the olfaction results and the fact that older people with olfactory impairment have been reported to be more than twice as likely to develop dementia after five years [24], there is a possibility that some of the “healthy” participants may be at risk for developing dementia. Thus, early identification of sensory decline in generally healthy older adults is essential from the perspective of health maintenance.

As for correlation between taste and olfaction, a trend was observed in the young group similar to the previous research [25], but no correlation was found in the older group. As it has been suggested that the correlation between taste and olfaction is a function of cognition occurring at the level of central processing [25], there may be a possibility of some error in cognitive processing in older participants, where the two senses are independent of each other.

In the current research, subjective health awareness was evaluated using simple questionnaires to invite intuitive answers, although the internal criteria might differ across ages. There was no significant difference between the young and older groups regarding subjective health awareness. It was revealed that although the poor results of the two sensory modalities indicate a decline in cognitive ability in the older group, the subjective health awareness of taste and olfaction in the older group was high. In addition, the older group showed an above-average level of self-confidence in their VAS score of overall health, and the score was comparable to that of the young group. These results suggest that the older people in the health promotion class may be satisfied with their state of health, and the contentment may have a positive influence not only on psychological wellness but also on physical health [10].

In this study, the relationship between sensory ability and subjective health awareness was tested only for young (around 20) and older adults (around 70). It may be of interest for future research to include other age groups to determine how the relationship between sensory ability and subjective health awareness changes over the course of an individual’s development. Specifically, the underlying mechanism of how the gap between sensory ability and subjective health awareness can be generated is worth exploring.
